# Fabrication of Micro- and Nanopillars from Pyrolytic Carbon and Tetrahedral Amorphous Carbon

**DOI:** 10.3390/mi10080510

**Published:** 2019-07-31

**Authors:** Joonas J. Heikkinen, Emilia Peltola, Niklas Wester, Jari Koskinen, Tomi Laurila, Sami Franssila, Ville Jokinen

**Affiliations:** 1 Department of Chemistry and Materials Science, Aalto University, Tietotie 3, 02150 Espoo, Finland; 2 Department of Electrical Engineering and Automation, Aalto University, Tietotie 3, 02150 Espoo, Finland; 3 Department of Chemistry and Materials Science, Aalto University, Kemistintie 1, 02150 Espoo, Finland

**Keywords:** pyrolysis, nanopillars, cell viability, ta-C, embossing, black silicon, neural stem cell, SU-8

## Abstract

Pattern formation of pyrolyzed carbon (PyC) and tetrahedral amorphous carbon (ta-C) thin films were investigated at micro- and nanoscale. Micro- and nanopillars were fabricated from both materials, and their biocompatibility was studied with cell viability tests. Carbon materials are known to be very challenging to pattern. Here we demonstrate two approaches to create biocompatible carbon features. The microtopographies were 2 μm or 20 μm pillars (1:1 aspect ratio) with three different pillar layouts (square-grid, hexa-grid, or random-grid orientation). The nanoscale topography consisted of random nanopillars fabricated by maskless anisotropic etching. The PyC structures were fabricated with photolithography and embossing techniques in SU-8 photopolymer which was pyrolyzed in an inert atmosphere. The ta-C is a thin film coating, and the structures for it were fabricated on silicon substrates. Despite different fabrication methods, both materials were formed into comparable micro- and nanostructures. Mouse neural stem cells were cultured on the samples (without any coatings) and their viability was evaluated with colorimetric viability assay. All samples expressed good biocompatibility, but the topography has only a minor effect on viability. Two μm pillars in ta-C shows increased cell count and aggregation compared to planar ta-C reference sample. The presented materials and fabrication techniques are well suited for applications that require carbon chemistry and benefit from large surface area and topography, such as electrophysiological and -chemical sensors for in vivo and in vitro measurements.

## 1. Introduction

Carbon materials are widely employed in many fields of science, from nanometer-scale transistors, flexible electronics, sensors, and batteries to carbon-composite metallurgy and carbon-fiber polymer composites, optics, and drug delivery systems. A carbon thin film was deposited for the first time in 1971 [1], and after that carbon has been an appealing coating material for reasons of wear and corrosion resistance, low friction, biocompatibility, hemocompatibility, electrical conductivity, and transparency [2,3,4]. In recent years it has found its way to medical devices (hip joints, stents, surgery tools, implants) [3,5,6,7,8] and life quality applications (biosensors) [8,9,10,11,12,13,14,15].

The spectrum of carbon materials is wide and not all are suitable for biosensor applications due to their synthesis methods, which can either leave harmful substances/materials on the film or the synthesis requires harsh conditions (e.g., high temperature can prevent the use certain processing steps). One of the promising materials with good biocompatibility is carbon attained through pyrolysis; a process which allows fabrication of complex carbon structures. The fabrication of pyrolytic carbon (PyC), especially from negative tone epoxy-based photoresist SU-8, is a well-established process [16], and the resulting material has showed great potential in biological applications [17,18,19]. The ability to choose the carbon substrate topography from several microns to as fine as single walled nanotubes has launched many studies related to cell survival and high surface area electrodes.

Diamond-like carbon (DLC) has emerged as a promising candidate for biomedical applications [20]. The mechanical and tribological properties together with biocompatibility, hemocompatibility, chemical inertness, and corrosion resistance makes the DLC materials unique [2]. DLC is not a specific single material, but a group of amorphous carbonaceous materials with different atomic bond structures and properties. Among DLC materials, tetrahedral amorphous carbon (ta-C) is a form with high sp^3^ bonding and low hydrogen content, and it is considered the hardest and most wear resistant [21], but it also possesses beneficial properties for sensor material: it is electrochemically active [13,15,22], it has a wide water window [23], it is transparent, and its deposition is possible at low temperatures [24].

Cells experience different environments, both chemical and physical, in the human body as they maturate during their lifespan. These environments shape the cell development and cell fate. Cells grown in vitro are typically cultivated on glass coverslips or petri dishes, which, in contrast to the complex three-dimensional topography of the human body, provide an inert and flat growth environment. Along with chemical messages received from adjacent cells, also the chemical composition of the surroundings plays a role. Materials such as glass and polystyrene (most common culture dish materials) do not provide any signals which promote (or hinder) the cell growth. To overcome this, there are plethora of materials and coatings which modify the surfaces to make them more cell compatible. In the ideal case, the substratum would provide suitable growth environment without the need for coatings. Many research findings show that different forms of carbon have this property [19,25].

The effects of microtopography on cell behavior have been studied extensively since 1964 when Curtis proposed that cells react to their geometrical environment [26]. Cells can react to topographical features as small as 5 nm [27], and even the conformation and symmetry of these nanofeatures may matter [28]. Most of the signaling factors that guide the cells into tissue assembly are not yet fully identified [29,30]. One clear and acknowledged signaling factor is contact guidance [30,31,32,33] which refers to the orientation of cells in influence of patterns of the substratum. The phenomenon is recognized, but the exact underlying biological events are still unknown. Many studies have shown that different topographies influence the cellular responses and resulted in changes in shape, differentiation and adhesion [34,35]. For example, all the functions of heart and cardiac cells (biochemical, electrical and mechanical) are uniquely dependent on their biological nanostructure [30,36]. Since the cells responds to both topographical and chemical cues from the surroundings, one critical questions has occurred repeatedly among the ones working in the field of scaffold fabrication for tissue engineering: does the method of fabrication of the physical topography actually change the surface chemistry and therefore create chemical patterns on the surface, which in turn might launch cellular reactions [32]. Despite the many studies, it remains unclear how cells react with the presence of conflicting physical and chemical cues [29,37].

Carbon materials are known for being challenging to pattern due to their corrosion resistance, hardness, and chemical inertness. Wet etching of carbon films is considered very challenging if not impossible as the internal carbon bonds are very strong. Different types of plasma etching processes are used with varying gas chemistries as the ion bombardment provides enough energy to break the carbon bonds and correct gas selection reacts with the resulting radicals [38]. Among the DLCs, the more sp^3^ hybridized carbon films are more challenging to etch compared to sp^2^ hybridized films.

Here we show fabrication processes for micro- and nanopillars from two different carbon materials, SU-8-based pyrolytic carbon (PyC) and tetrahedral amorphous carbon (ta-C) for applications that use carbon chemistry and benefit from large surface area and topography. The presented micro- and nanotopographies are pillars in three different scales: 20 μm, 2 μm, and <1 μm. We also study the growth of mouse neural stem cells when the carbon substratum is micro- and nanopatterned.

## 2. Experimental

**Figure 1:**
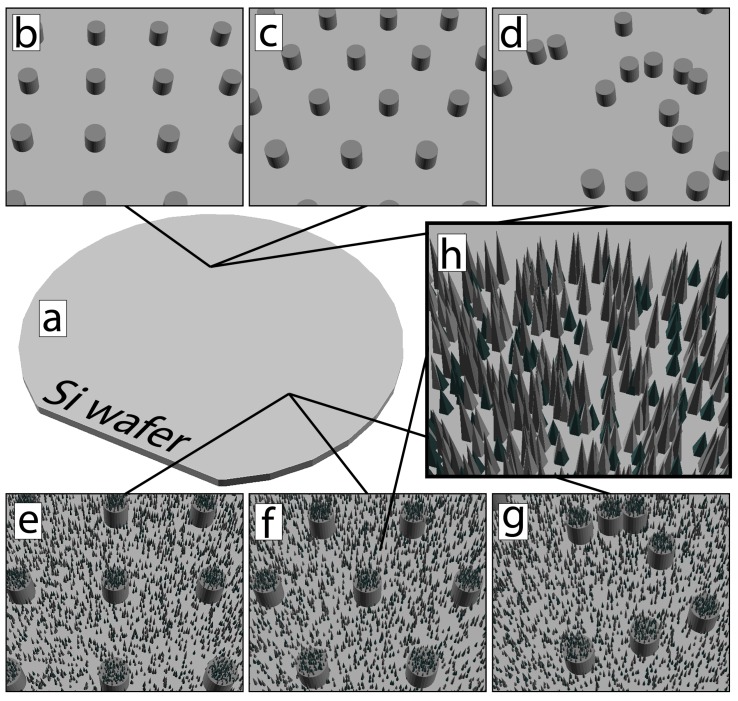
Schematic structures of studied micro- and nanotopography. (**a**) Planar silicon surface coated with either material. Microtopography with 2 μm or 20 μm (1:1 aspect ratio) pillars in (**b**) square-grid layout, (**c**) hexa-grid layout or (**d**) random-grid layout, either silicon coated with tetrahedral amorphous carbon (ta-C) or made entirely of pyrolytic carbon (PyC). (**e**–**g**) Same pillars but now nanoroughened with black silicon structures, so-called “dual-scale” structure. (**h**) Nanoroughness in the form of black silicon structures (small pyramid-like spikes in random pitch and size).

Two different carbon substrates, PyC and ta-C, were fabricated with different micro- and nanoscale topographies. Samples with ta-C were fabricated by etching and thin film deposition, whereas PyC samples also used UV-embossing techniques [39,40]. The PyC samples were fabricated with 11 different combinations of micro- and nanotopography, and ta-C samples with eight different combinations. The microtopography was either 2 μm or 20 μm pillars, aspect ratio 1:1, and the layout of the pillars was either square-grid, hexa-grid or random-grid. Nanotopography was either native planar (no specific treatments to modify the roughness) or nanopillars (“black silicon”, bSi) [41]. Figure 1 shows the schematics of different combinations studied.

### 2.1. Pyrolyzed Carbon Sample Fabrication

The planar reference, 2 μm and 20 μm pillar structures for pyrolyzed carbon samples were fabricated by direct photolithography of SU-8. The nanopillar and dual-scale pillar structures were fabricated by UV-embossing technique, which required a silicon master and a PDMS (polydimethylsiloxane) stamp to be fabricated first. All SU-8 structures were pyrolyzed in the same conditions.

#### 2.1.1. Silicon Master

**Figure 2:**
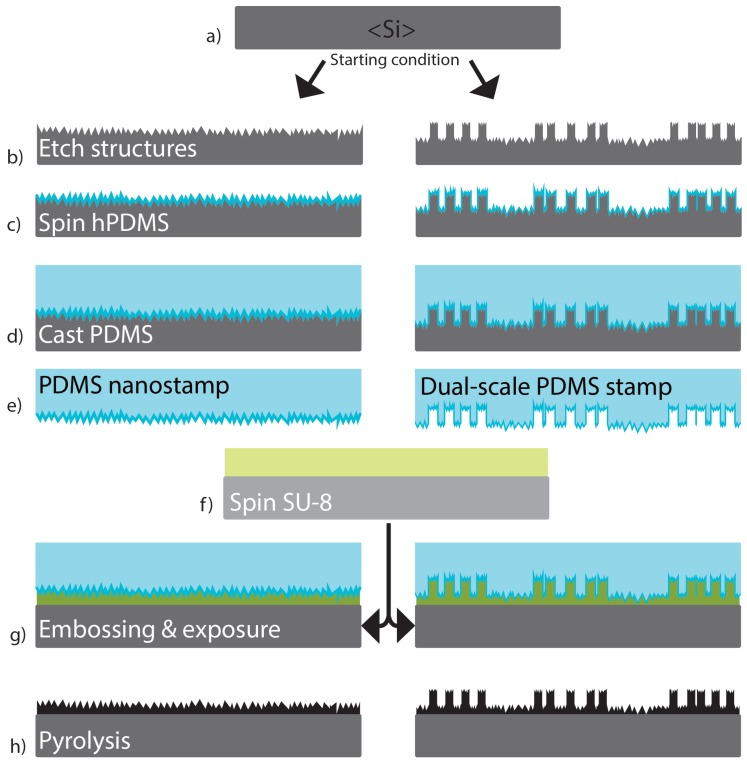
Process flow for pyrolytic carbon micro- and nanotopography fabrication with UV-embossing. First, (**a**) a planar silicon wafer was patterned either with (**b**) nanoroughness, or with micropillars and nanoroughness. Then (**c**) hard-polydimethylsiloxane (h-PDMS) was spun on the master, (**d**) normal 10:1 PDMS was cast on top of it and (**e**) solid hPDMS-PDMS stamps were peeled off. (**f**) SU-8 was spun on another silicon wafer where (**g**) the stamps were embossed and exposed to UV-light. (**h**) After peeling of the stamps the patterned SU-8 layers were pyrolyzed.

The black silicon (bSi) masters were fabricated with maskless anisotropic silicon etching in ICP-RIE (Inductively Coupled Plasma Reactive Ion Etching, Plasmalab 100—ICP 180, Oxford Instruments, Abingdon, UK) equipment with a process described by Sainiemi et al. [40]. No pre-treatments were done for silicon wafers before etching. The etching recipe was: SF_6_ flow 40 sccm, O_2_ flow 18 sccm, pressure 10 mTorr, temperature −110 °C, RF-power 6 W, ICP-power 1000 W, etch time five minutes (Figure 2b left).

The dual-scale 2 μm pillar–bSi samples were fabricated by first etching the pillars with ICP-RIE by masking the pillar parts, and then using the maskless anisotropic silicon etching to form the bSi structures on top of and between the pillars (Figure 2b right). First, a 100 mm silicon wafer <100> was covered with 50 nm sputtered chromium (Plasmalab 400, Oxford Instruments). Then the wafer was treated with HMDS (hexamethyldisilazane) to improve photoresist adhesion. Then a photoresist AZ5214 (MicroChemicals, Ulm, Germany) was spin coated on the wafer in 4000 rpm for 30 s, followed by soft baking on a hotplate of 90 °C for two minutes. Then the baked resist was exposed to UV-light (365 nm, SUSS Mask aligner MA-6, SUSS MicroTec, Garching, Germany) for three seconds and developed in diluted AZ351B (MicroChemicals) developer for one minute (dilution 1:5 AZ351B:DIW). Finally, the resist was hard baked on a hotplate at 120 °C for three minutes to remove remaining solvent.

The patterned resist was used as an etching mask for Cr etching. The chromium layer was etched through AZ5214 mask in 3:1:21 Ce(NH_4_)_2_(NO_3_)_6_:HClO_4_:H_2_O solution (MicroChemicals) in room temperature approximately 90 s and rinsed with DI-water for five minutes and dried with a nitrogen gun. The resist was removed in an ultrasound assisted acetone bath for 10 min, then rinsed with clean acetone and isopropyl alcohol and finally with DI-water, followed by spin-drying.

Chromium was used as a hard etching mask for ICP-RIE silicon etching. The used recipe was: SF_6_ flow 40 sccm, O_2_ flow 6 sccm, pressure 8 mTorr, temperature −110 °C, RF-power 3 W, ICP-power 1000 W, etch rate 1.3 μm/min, target depth 2 μm. After etching the chromium layer was removed in Cr etchant, rinsed with DI-water and dried with spin-drying and in oven at 120 °C for 30 min. Finally, the nanopillars were etched on the micropillars with the same recipe as the bSi stamp master was made. The heights of the pillars were measured with Bruker Dektak XT stylus profilometer (Bruker, Billerica, MA, USA).

Both wafer types (bSi and dual-scale pillar-bSi) were coated with fluoropolymer (Oxford Instruments, Plasmalab 80Plus) to prevent PDMS stamp adhesion on the surface.

#### 2.1.2. hPDMS-PDMS Stamp

The PDMS stamps were fabricated with dual-layer process: first, a thin layer of hard-PDMS (hPDMS) was spin coated on the surface of fluoropolymer-coated silicon wafer and then normal 10:1 PDMS was cast to provide a bulk structure of the stamp. The hPDMS layer was fabricated by first mixing 3.4 g of vinyl PDMS prepolymer ((vinylmethylsiloxane)-dimethylsiloxane copolymer), 17.5 μL platinum catalyst (platinum divinyltetramethyldisiloxane), and 8 μL of modulator (1,3,5,7-tetravinyl- 1,3,5,7-tetramethylcyclotetrasiloxane). The mixture was degassed for five minutes and then the last component, 1 g of hydrosilane prepolymer ((methylhydrosiloxane)-dimethylsiloxane copolymer), was added. The addition of the last component starts the polymerization process and there was approximately 10 min time to spread the hPDMS mixture on the substrate, and the amount was sufficient for single 100 mm wafer. The liquid hPDMS mixture was spin coated on the stamp masters with 1000 rpm for 20 s (Figure 2c). After spin coating, 10:1 PDMS was cast on top of the mixture to thicken the stamp (Figure 2d). This had to be done while the hPDMS is still in liquid form. Air bubbles were degassed from the mixture in a vacuum desiccator. After degassing, the hPDMS-PDMS liquid stamps were baked in an oven at 50 °C for two hours. After baking the solid stamps were cut around the wafer and peeled off (Figure 2e).

#### 2.1.3. SU-8 Processing

The SU-8 processing was done on highly boron doped 100 mm silicon wafer (p++, <100>, Siegert Wafer, Aachen, Germany). First the wafer was immersed into BHF (buffered hydrofluoric acid) to remove native oxide from the wafer surface. Then the wafer was rinsed with deionized (DI)-water, dried by spin-drying and moisture was removed in an oven at 120 °C for five minutes. Then negative tone photoresist SU-8 was spin coated on all sample types. Following paragraphs describe the fabrication of the different samples in detail.

**Planar reference**

The reference sample has no structures or features on it. The wafer was spin coated with SU-8 50 (9000 rpm, 45 s, thickness aim for 13 μm), followed by a ramped soft bake (ramp up 15 °C/min to 65 °C, hold three minutes, ramp up 15 °C/min to 95 °C, hold five minutes, cool down naturally). The solidified polymer film was flood exposed to UV-light for eight seconds, followed by a ramped post-exposure bake (PEB) (ramp up 15 °C/min to 95 °C, hold four minutes, cool down 3.75 °C/min to room temperature).

**2 and 20 μm pillars**

The micropillar samples had two layers of SU-8: a base layer and a pillar layer. First, the wafers were spin coated with SU-8 5 (5000 rpm, 30 s, thickness aim for 3 μm for base layer), followed by a ramped soft bake (same as with reference). The solidified polymer film was flood exposed to UV-light for five seconds, followed by a ramped PEB (same as with reference). The pillar layer was spin coated with SU-8 5 for 2 μm pillars (5000 rpm, 40 s, thickness aim for 2 μm) and with SU-8 50 for 20 μm pillars (9000 rpm, 30 s, thickness aim for 20 μm). The 2 μm pillar wafer was soft baked similar to reference and the 20 μm pillar wafer was soft baked by ramping the temperature (ramp up 15 °C/min to 65 °C, hold five minutes, ramp up 10 °C/min to 95 °C, hold eight minutes, cool down naturally). The polymer layers were exposed to UV-light through a photomask containing either 2 μm openings or 20 μm openings: SU-8 5 for five seconds and SU-8 50 for eight seconds. Post exposure bake (PEB) was also ramped: ramp up 15 °C/min to 95 °C, hold four minutes for SU-8 5 and eight minutes for SU-8 50, cool down 3.75 °C/min to room temperature for both SU-8 polymers. Both sample types were developed in mr-Dev 600 (micro resist technology GmbH, Berlin, Germany) developer to remove unexposed resist. Development time was five minutes for SU-8 5 and 10 min for SU-8 50. Both samples were rinsed with isopropyl alcohol and DI-water, and dried in an oven at 90 °C for 10 min.

**Nanorough and dual-scale**

Black silicon (bSi) and dual-scale pillar-bSi samples were fabricated with UV-embossing technique. Both wafer types were spin coated with SU-8 50 (9000 rpm, 45 s, thickness aim for 13 μm) and soft baked with ramping (ramp up 15 °C/min to 65 °C, hold three minutes, ramp up 15 °C/min to 95 °C, hold five minutes, cool down naturally until temperature is at 75 °C or 65 °C and then stop cooling). While the substrate was in 75/65 °C, the hPDMS-PDMS stamp was placed on top of the soft SU-8 and squeezed tightly, making sure no air bubbles are left between the stamp and the substrate. When the stamp is firmly in place, cool down was continued until room temperature was reached. After this the whole substrate-stamp combination was flood exposed to UV-light for 16 s to start the polymerization process in SU-8 (Figure 2g). PEB was done similar to reference sample. The stamps were gently peeled off in room temperature, making sure no hPDMS is left on the surface.

#### 2.1.4. Dicing

The dicing was done with DISCO DAD3220 dicing saw (DISCO Corporation, Tokyo, Japan). Before dicing, all other samples than reference sample were protected with AZ5214 photoresist during dicing (spin coating 500 rpm for 20 s, soft bake 90 °C for 5 min, no exposure or hard bake). The diced sample size was 10 mm × 10 mm. After dicing, the chips were washed twice in acetone to remove protecting photoresist, rinsed with isopropyl alcohol and DI-water, and dried with nitrogen gun and in an oven at 70 °C for 30 min.

#### 2.1.5. Pyrolysis

The pyrolysis process was carried out in a Nabertherm RS 80/500/11 horizontal tube furnace (Nabertherm GmbH, Lilienthal, Germany). First, the samples were placed into ceramic carrier boats, and the boats were inserted in the middle of the tube furnace where the temperature gradient was most stable. The tube was pumped down to pressure of 4 mbar and flushed with nitrogen three times to remove most of the oxygen inside. After last flush, low nitrogen flow was left on and the inside pressure was kept at atmospheric pressure. The first step of the pyrolysis was to heat the furnace to 300 °C and hold it for 40 min to completely eliminate the remaining solvent and unreacted monomers from the film [16]. Then the temperature was ramped to 900 °C and held for 60 min to start the actual pyrolysis process during which most of the oxygen, nitrogen and hydrogen are eliminated from the film and aromatic network becomes interconnected (Figure 2h). Then the furnace was slowly (for 12 h) cooled down to room temperature. The ramp up rates during heating were 200 °C/h. The heights of the pillars were measured with Bruker Dektak XT stylus profilometer.

### 2.2. ta-C Sample Fabrication

**Figure 3:**
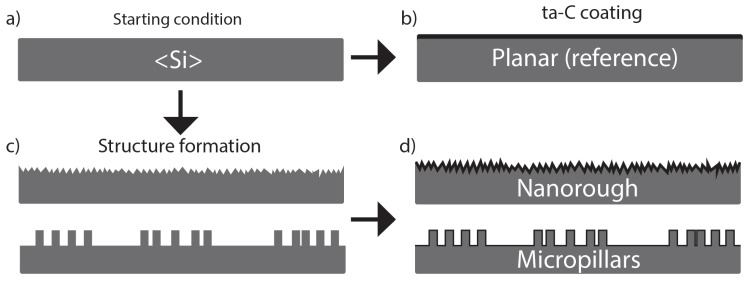
Fabrication process flows for ta-C samples. (**a**,**b**) For planar samples, ta-C was deposited directly on silicon wafer without any treatments. (**c**) For nanorough samples, the silicon wafer was anisotropically etched to produce random pyramid-like roughness on the surface before ta-C deposition. For pillars (three different layouts, two sizes) the silicon wafer was etched with patterned Cr hard mask on the surface. (**d**) After structuring, the ta-C was coated on the surface.

Samples with ta-C were fabricated by coating highly boron doped 100 mm silicon wafers (p++, <100>, Siegert Wafer) with or without structures. The process flow for all ta-C samples is presented in Figure 3. The reference samples were created from silicon wafers without any treatments or features (planar, Figure 3a,b). The black silicon (bSi) wafers were fabricated with maskless anisotropic silicon etching using ICP-RIE with a process described by Sainiemi et al. [40] (Figure 3c). No pre-treatments were done for silicon wafers before etching. The etching recipe was: SF_6_ flow 40 sccm, O_2_ flow 18 sccm, pressure 10 mTorr, temperature −110 °C, RF-power 6 W, ICP-power 1000 W, etch time seven minutes.

The silicon pillar samples were created with the same process as the silicon masters for UV-embossing. The pillar etching in ICP-RIE had target depth either 2 μm or 20 μm. The 7 nm ta-C coating (without adhesion layers) for all wafers was deposited with pulsed filtered cathodic vacuum arc (p-FCVA) (Figure 3b,d). In p-FCVA the material from cathode target is vaporized with an electric arc. The plasma is then directed with coils to a substrate where it forms a thin film with high sp^3^ fraction. In addition to the carbon plasma the explosive emission process creates macroparticles that are filtered out with a 45° bent magnetic filter. The deposition process is described in greater detail in [42].

Finally, the samples were diced with DISCO DAD3220 dicing saw into 10 mm × 10 m pieces. The samples were cleaned with isopropyl alcohol and DI-water immersion to remove dicing residues.

### 2.3. Cell Cultures

For cell culture experiments, the samples were sterilized in 70% ethanol in petri dishes for 10 min, after which most of the ethanol was removed, followed by evaporation for 20 min.

Cells were cultured in humidified incubator with 5% CO_2_ in the air. Mouse neural stem cells (mNSC, ATCC^®^ CRL2926™, Manassas, VA, USA) were cultured in Eagle’s Minimum Essential Medium supplemented with 2 mM L-Glutamine, 10% fetal bovine serum, 100 IU/mL of penicillin and 100 μg/mL of streptomycin. The mNSCs were seeded at 30,000 cells/cm^2^. The cells were cultured on samples placed on 12-well plates for 24 h.

The viability rate of cells was tested by 3-(4,5-dimethylthiazol-2-yl)-2,5-diphenyltetrazolium bromide (MTT) assay. After culture, the samples were transferred to a clean 12-well plate and 0.5 mg/mL of MTT was added in the colorless medium. After 3–4 h incubation at 37 °C in a humidified chamber, MTT was dissolved by adding 1:4 20% sodium dodecyl sulfate (Sigma Aldrich, St. Louis, MO, USA) in 0.02 M HCl. Samples were incubated overnight, and 800 μL of the media was transferred to a clean 48-well microplates and the absorbance was read with an automated plate reader at 570 nm (FLUOstar Optima, Ortenberg, Germany). Data was collected from triplicate samples. Viability was compared to planar ta-C or PyC (100% viability).

For actin staining, the cells were fixed in 4% paraformaldehyde (PFA) for 15 min. Cells were stained using phalloidin-568-label (Biotium 1:50, 30 min incubation) and nuclei by DAPI (Vectrashield mounting medium with DAPI). Olympus BX51M microscope and Leica DCF420 digital microscope camera were used for the imaging.

## 3. Results and Discussion

### 3.1. PyC Structures

We fabricated 2 μm and 20 μm pillar structures with direct photolithography and nanopillar and dual-scale pillar structures with UV-embossing techniques followed by pyrolysis of SU-8.

#### 3.1.1. Black Silicon Profile Optimization for Replication

**Figure 4:**
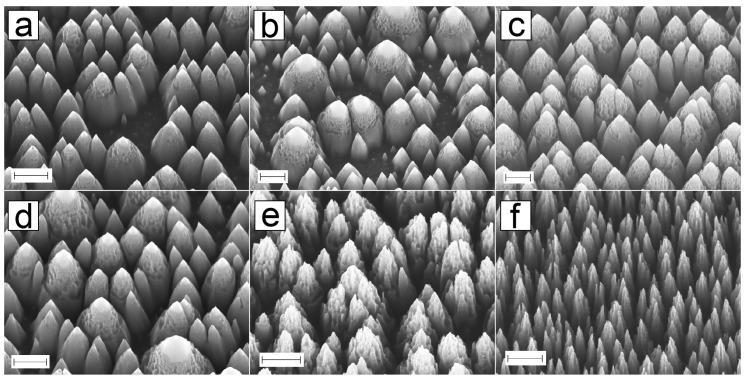
Scanning electron microscope (SEM) images of black silicon structures with variable etching times. Anisotropic etching profile creates sharp pyramid-like structures on silicon. The structures become denser when the etching time is increased from (**a**) 3.5 min to (**b**) 3.75 min. Almost all planar silicon is consumed with etching time of (**c**) 3.875 min. Structure size stays approximately the same when the etching time is (**d**) 4 min, but the surface roughness increases which is clearly visible in (**e**) 5 min. After (**f**) 7 min of etching, the pillar size, density, and roughness reach the maximum surface area. All scalebars: 1 μm

Figure 4 shows the evolution of nanopillar formation with increased etching time. The previously reported black silicon (bSi) etching process (7-min etching time) [40] creates random sharp and rough nanopillars, as shown in Figure 4f. The name “black silicon” comes from the black visual appearance of the surface resulting from the roughness. The cast hard-polydimethylsiloxane (h-PDMS) attached strongly on the nanopillars, therefore after curing the adhesion on the substrate was stronger than cohesion inside hPDMS-PDMS and because of this the process was unreliable and frequently led to breaking of the replica. Due to this we optimized the black silicon process for replication. First the etching time was varied between 2, 3, 4, 5, 6, and 7 min. We found out that first critical change in bSi appearance occurs between 3 and 4 min (Figure 4a,d): the density of pillars after 3 min is relatively low, as there is still planar silicon present in the wafer, whereas these planar areas are not recognizable after 4 min of etching. A second etching series focused between these two timepoints. Figure 4a shows a SEM image of a sample etched for 3.5 min, and the planar areas can still be seen. Therefore, the change happens somewhere between 3.5 min and 4.0 min. For the second series, we divided the critical range into 3 timepoints: 3.625, 3.750, and 3.875 min. From the scanning electron microscope (SEM) images (Figure 4a–c for 3.5 min, 3.750 min and 3.875 min, respectively) we found out that the density increased around etching time of 3.875 min (Figure 4c). Between 4 and 5 min of etching, the pyramid size stays approximately the same, but the surface roughness increases (Figure 4d,e). After 7 min of etching, the structures acquire the sharp with needle-like topography (Figure 4f). Throughout the whole etching procedure, the process seems to first form big, 1 μm size bumps, which then turn into pillars (Figure 4a). When the etch is continued, the pillars get higher, denser (Figure 4c), and a rough “cap” is formed on the tip (Figure 4d). The cap acts as a starting point for new etch planes and after 5 min of etching (Figure 4e) the cap is roughened even more, and the pillar size is decreased. Some pillars are split into two or more. After 7 min of etching (Figure 4f) the maximum density is reached and the nanopillars are sharp needles with some roughness on the sidewalls. When the etch is continued for 21 min, the density of pillars stays approximately the same, but the sidewall roughness is lost.

From these tests we could conclude that the roughness and density of nanopillars is high after 5 min of etching, but the nanopillars have positive sidewalls and therefore it was chosen as the ideal profile for embossing. All subsequent experiments used the 5 min etching time for black silicon unless otherwise specified.

#### 3.1.2. Dual-Scale Micro- and Nanopillars

**Figure 5:**
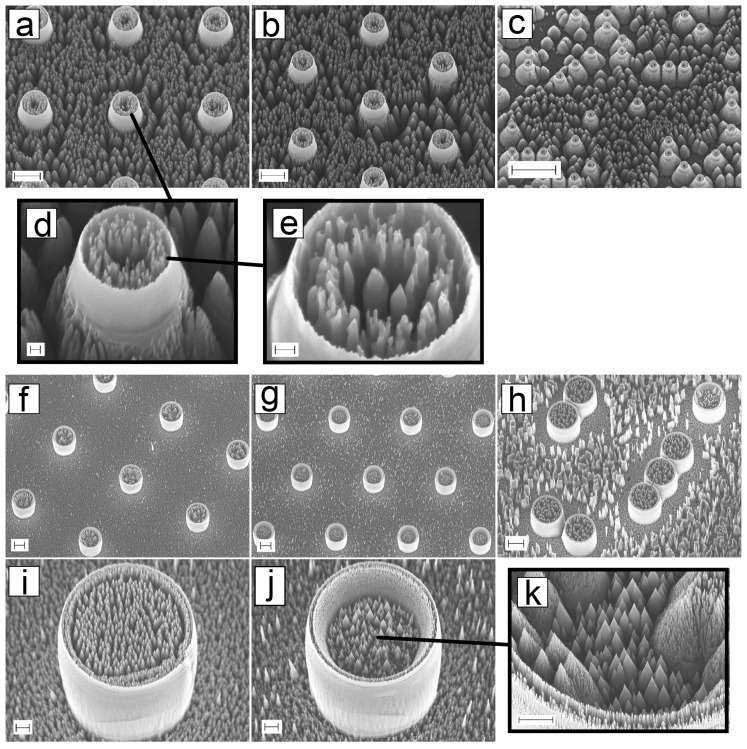
SEM images of silicon master for hPDMS-PDMS molding. 2 μm pillars etched for 5 min in order to create bSi roughness with (**a**) square-grid (scalebar: 2 μm) (**b**) hexa-grid (scalebar: 2 μm) and (**c**) random-grid (scalebar: 10 μm) layout. (**d**) Closeup of an individual pillar shows that the inside of the pillar is made hollow (scalebar: 200 nm). (**e**) Closer view shows the size of the roughness (scalebar: 200 nm). 20 μm pillar in different layout; (**f**) square-grid (scalebar: 10 μm), (**g**) hexa-grid (scalebar: 10 μm) and (**h**) random-grid (scalebar: 10 μm). The bSi etch for 20 μm pillars created two kinds of structures: (**i**) pillar with roughened surface (scalebar: 2 μm) and (**j**) pillar as a “cup” (scalebar: 2 μm). (**k**) The bottom of the “cup” structure had sharp pyramids (scalebar: 2 μm).

Figure 5a–c show SEM images of nanoroughened 2 μm pillars with square-grid, hexa-grid, or random-grid layouts. From Figure 5a,b we can see that the bottom surface between pillars is roughened, but the pillar height has not changed much. From Figure 5c we can see the effect of random nature of the etching process: some pillars are only etched deeper and no roughness is present between the pillars. Figure 5d and e show a closer image of an individual pillar. From these, we can see that the roughness is also present inside the pillar, and the sidewalls are still intact. Therefore, we have acquired the nanoroughness both between and on top of the pillars.

The 20 μm pillar structures are shown in Figure 5f,g, and h in three different layouts. The process time for black silicon etching was 7 min for the 20 μm dual-scale pillars. Figure 5h shows some bigger nanostructures present between the pillars. From the individual pillars, mainly two types of profiles could be found: Figure 5i shows a pillar where the roughness is at the same level as the pillar top is and Figure 5j shows a pillar where the bSi etch goes inside the pillar, thus creating a rough “cup”. Figure 5k shows a closer image of an individual pillar where both types of profiles are present. From these dual-scale pillar-bSi structures, we used only the 2 μm pillar-bSi as a mold for SU-8 embossing.

#### 3.1.3. SU-8 Embossing

For embossing, we used two kinds of stamps: stamps with bSi structures and stamps with dual-scale 2 μm pillar-bSi structures. During detaching the stamps from silicon master, some cracking lines could be identified in the hPDMS layer, but these crack lines did not copy onto the SU-8. The PDMS layer on top of hPDMS had to be at least 2 mm thick to provide robustness to the stamp.

The structures from the mold had to be copied on SU-8 while it was still above glass transition temperature (50 °C before exposure [43]). We first used temperature of 65 °C but after detaching the hPDMS-PDMS stamp, we saw that only 50% of the SU-8 had copied the bSi structure. Therefore, we did the same procedure with 75 °C, and saw a major increase (to approximately 90%) in the copied surface, and this was used in all subsequent embossing steps. The temperature did not have major impact on the shape of copied bSi structures and separating the mold from SU-8 was as easy in both temperatures.

#### 3.1.4. Pyrolysis

**Figure 6:**
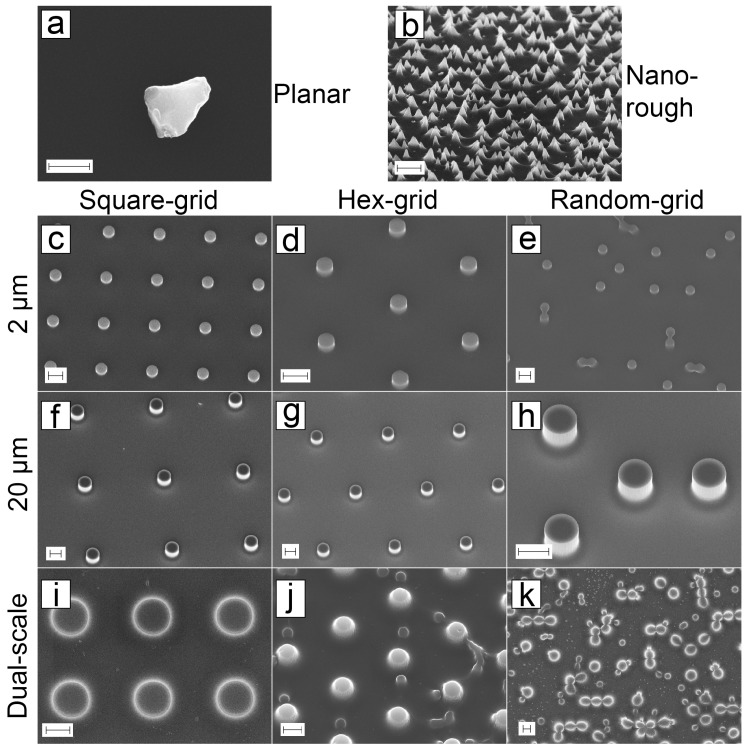
SEM images of PyC structures made with direct lithography (**a**), (**c**–**h**) and UV-embossing (**b**), (**i**–**k**). (**a**) Planar reference sample with silicon particle on the surface to show the absence of structures (scalebar: 1 μm). (**b**) PyC nanopillars made with embossing (scalebar: 2 μm). All pillar structures with three layouts: (**c**–**e**) 2 μm pillars (scalebar: 2 μm), (**f**–**h**) 20 μm pillars (scalebar: 10 μm) and (**i**–**k**) Dual-scale pillars (scalebar: 2 μm).

SEM images of the PyC structures are presented in Figure 6. The planar PyC sample can be seen in Figure 6a with a silicon dust particle to help focus on the surface. No features can be observed on the reference sample. The bSi structure copied on PyC is presented in Figure 6b. Figure 6c–e shows SEM images on 2 μm pillars which were created with direct photolithography, as well as the 20 μm pillars in Figure 6f–h. The pillar-bSi structures are viewed in Figure 6i–k.

The replication process for PyC nanopillars in Figure 6b was successful in terms of nanoroughness size. The individual pyramids are <1 μm in size and the height and density is lower than in the silicon master. This is probably due to the SU-8 shrinkage during pyrolysis.

**Figure 7:**
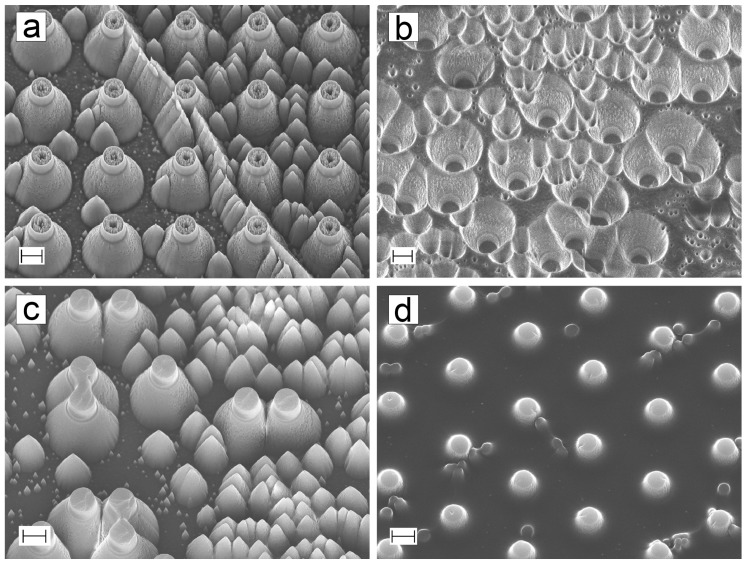
Carbon micro- and nanopillar fabrication by embossing and pyrolysis. (**a**) Dual-scale pillar-bSi structures had two kinds of topography: either the interpillar space had only a few nanopillars (left), or it was full of them (right). (**b**) The hPDMS-PDMS stamp. (**c**) The copy process into SU-8 layer was also successful with even the smallest of features. (**d**) Dual-scale pillar-bSi structures after pyrolysis. All scalebars: 2 μm

The dual-scale structure should contain the nanorough surface as well as the pillars, but as can be seen from the Figure 6i, there are only few nanopillars present. The area between the pillars has some roughness, but not as much as the PyC-bSi sample (Figure 6b). This issue was inspected further, and SEM images of all embossing steps are presented in Figure 7. The random nature of nanopillar formation can be seen in Figure 7a where the nanopillars are formed only between some of the pillars. Nanopillars were present on top of the micropillars. These structures were copied in high detail into hPDMS-PDMS stamp, except for the nanopillars on top of the micropillars (Figure 7b). This might be due to air being trapped inside the hollow pillars. The copy process was also successful on SU-8 (Figure 7c) where all the features from hPDMS-PDMS stamp can be recognized. The last step was pyrolysis of the SU-8, during which the nanopillar roughness was mostly lost. The pyrolysis process shrinks all dimensions, and this probably has a major effect on the nanopillars. The effect is not as drastic with plain PyC-bSi (Figure 6b), possibly because there are more nanopillars present in the silicon master compared to pillar-bSi master.

#### 3.1.5. Shrinkage

During pyrolysis process, the SU-8 polymer loses oxygen atoms from the reacted epoxide-groups which results in new chemical arrangements of carbon atoms. Since the crosslinked SU-8 contains a lot of -OH groups, the mass loss is significant during pyrolysis and structural shrinkage occurs. This shrinkage affects to all dimensions in the structures. Based on our studies with pyrolyzed structures (unpublished data), the shrinkage is strongest in z-direction (thickness), and in xy-direction the shrinkage is stronger with larger structures. In this work the 2 μm pillars shrunk 50% in height and 25% in diameter. The 20 μm pillars shrunk 50% in height and 40% in diameter. The pillars in dual-scale structures shrunk 80% in height and 30% in diameter.

### 3.2. ta-C Structures

**Figure 8:**
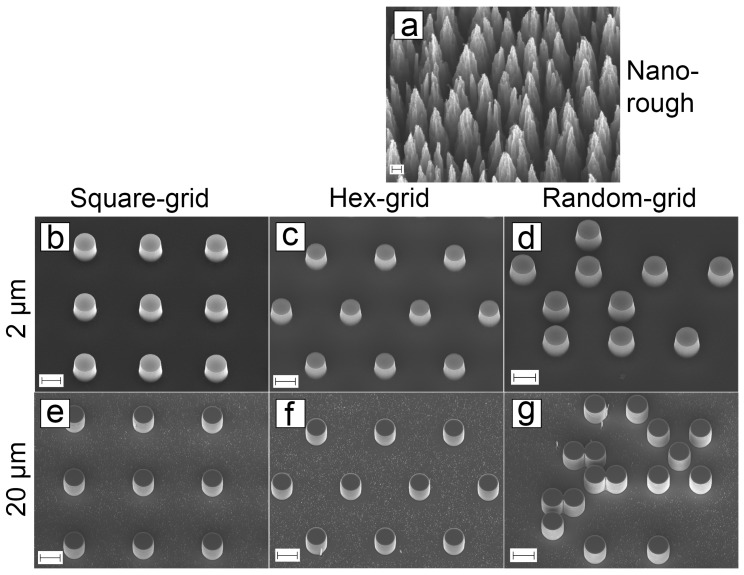
SEM images of ta-C coated silicon structures. Planar sample is not shown here as it was too smooth to focus on with SEM. (**a**) Nanorough silicon coated with ta-C (scalebar: 200 nm). Silicon pillars coated with ta-C in three different layouts: (**b**–**d**) 2 μm pillars (scalebar: 2 μm) and (**e**–**g**) 20 μm pillars (scalebar: 20 μm).

Eight different nano- and microrough surfaces were fabricated with silicon etching and thin film ta-C deposition: planar, nanopillars, 2 μm pillars and 20 μm pillars in three different layouts. SEM images from the structures are shown in Figure 8.

The reference sample was a planar silicon wafer coated with ta-C. The filtered cathodic vacuum arc (FCVA) deposited ta-C films are typically ultrasmooth [44] and atomic force microscope (AFM) measurements have shown that ta-C films deposited with the same equipment produces coatings with root mean square (RMS) surface roughness of 0.1–0.2 nm [23] and this coating copies the roughness of underlying substrate in few nm roughness range [24].

The black silicon structure for ta-C coating was etched with 7-min recipe (Figure 8a). The 2 μm pillars were created with ICP-RIE process with chromium as a hard etching mask (Figure 8b–d). The actual heights of the pillars were 2.2 μm. The minor change in depth is due to inaccuracies during etching process and timing. 20 μm pillars were created with similar process, but with longer etch time (Figure 8e–g). The actual heights of the pillars were 21.7 μm.

Figure 8b–g show that the sidewalls have a positive profile (anisotropic etch profile). This allows the ta-C deposition to also occur to some extent on the sidewalls, therefore the grown cells detect mostly ta-C around them.

### 3.3. Fabrication Comparison

Structures made from pyrolyzed carbon are made entirely from the same material, whereas ta-C processing is a coating for existing structures. There are advantages and disadvantages in both methods. The benefits for PyC processing are the direct use of photolithography for structure fabrication and cheaper procedures. Great advantage is the possibility to create overhangs and suspended structures with lithography which will be pyrolyzed in the end to get high surface area 3D carbon microstructures. The PyC process creates micro- and nanopillar structures that are wholly made of carbon, which enables their use for applications that use bulk properties such as conduction and absorption. Furthermore, bulk structures are not susceptible to adhesion issues that can be a problem for thin film coatings. The UV-embossing technique requires more complex fabrication although the silicon and hPDMS-PDMS masters are reusable. This fabrication procedure is well suited for lower grade laboratories without daily access to high-grade cleanroom with etching equipment for silicon processing. Downsides in PyC processing are the shrinkage during pyrolysis (if it is not taken carefully into account during process planning), accuracy in structure dimensions and high temperature processing. In comparison, ta-C is deposited in room temperature, the underlying structures are made of silicon which has well known processing procedures and therefore the control of the fabricated structures is better. However, p-FCVA provides line-of-sight film conformality and therefore with suspended structures only the top will be coated. In addition to lithography, fabrication of every sample requires deposition and etching tools, making this kind of fabrication more suitable for facilities which have proper high-end processing tools readily available.

PyC microstructures have been made in a study by Amato et al. [18] where they use pyrolyzed carbon micropillars for stem cell differentiation and electrochemical detection of dopamine. In that study the resulting PyC pillar diameter was 1.4 μm and height 11 μm, which are in same size scale as our pillars, but different aspect ratio. They report that human neural stem cells adhere poorly on their untreated (no PLL or oxygen plasma treatment) PyC surfaces, whereas we had good cellular adhesion on our untreated surfaces. Many other studies have used pyrolyzed carbon for plethora of different applications, such as DNA detection [45], supercapacitors [46], neural cell differentiation [47] and glucose detection [48]. Jiang et al. [46] created the micro- and nanotopography in their study with supercapacitors. Micropatterns were created with positive resist AZ9260 and with embossing methods the structures were copied to SU-8 layer and it was pyrolyzed. The nanoroughness was acquired with oxygen plasma etching before pyrolysis. The resulting nanowires were in same scale as our nanopillars, but their density was not as high.

There are numerous studies which have created nanopillar structures on silicon (called black silicon), but only a few has used carbon coating on it to further enhance the usage possibilities. Shah et al. [49] created a black silicon surface and coated it with CVD pyrolyzed carbon to decrease the surface reflectance. The PyC thickness was 25 nm, and the coating method preserved even the smallest details, keeping the pillars sharp. May et al. [50] coated similar nanopillars with detonation nanodiamond (DND) which increased the pillar diameter and made them blunt. This surface was used in the electrochemical detection of dopamine and as an antibacterial surface. Similar bactericidal activity of black silicon surfaces has been reported by other research groups as well [51,52]. The bactericidal activity is due to cell membrane rupture, stretching, or critical deformation, and gram-positive and gram-negative bacteria behave differently [53,54,55]. Fisher et al. [56] fabricated diamond nanocone surfaces with cone diameter in the same scale as our silicon nanopillars, and they also report antibacterial properties on their surfaces. This lets us conclude that also our topography should be enough to affect cell fate.

### 3.4. Cell Compatibility

#### 3.4.1. Cell Cultures on PyC

**Figure 9:**
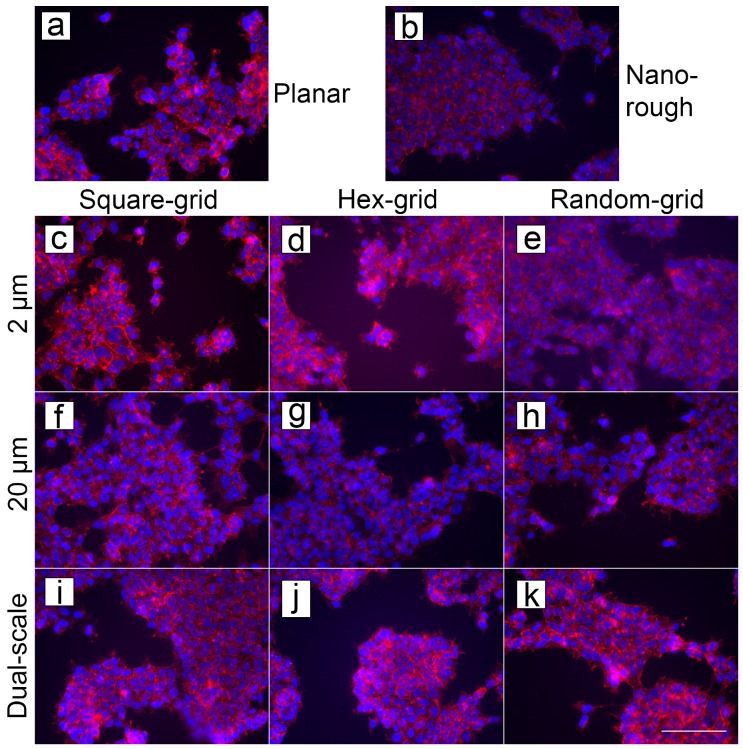
Stained mNSC on PyC structures. Cells on (**a**) planar PyC substrate (reference), (**b**) nanopillar structures copied in PyC, (**c**–**e**) 2 μm pillars, (**f**–**h**) 20 μm pillars and (**i**–**k**) dual-scale pillars. Scalebar: 100 μm

Mouse neural stem cell (mNSC) cultures on PyC samples are shown in Figure 9. MTT showed no statistically significant differences on the surfaces. The planar PyC is used as a reference (Figure 9a).

#### 3.4.2. Cell Cultures on ta-C

**Figure 10:**
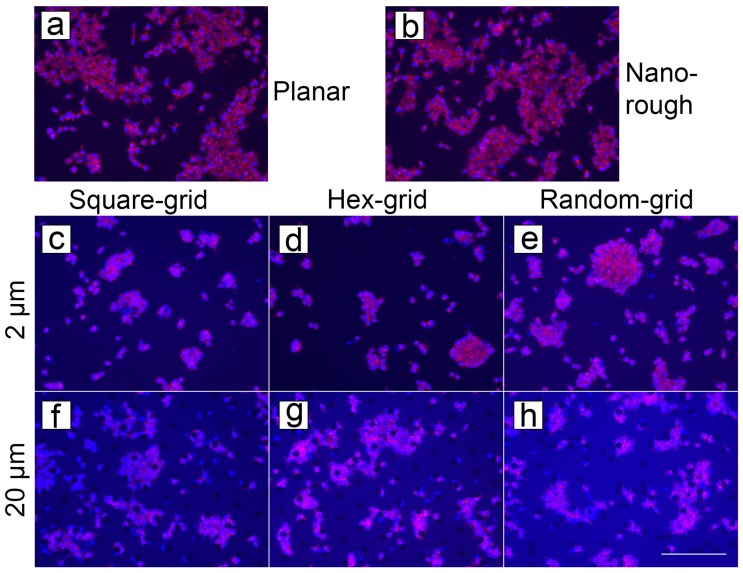
Stained mNSC on ta-C structures. Cells on (**a**) planar ta-C as a reference, (**b**) nanorough silicon surface with ta-C coating, (**c**–**e**) 2 μm pillars and (**f**–**h**) 20 μm pillars. Scalebar: 200 μm

Mouse neural stem cell cultures on ta-C samples are shown in Figure 10. MTT indicated higher cell viability on 2 μm pillars (Figure 10c–e) compared to planar surface (Figure 10a) or 20 μm pillars (Figure 10f–h). The nanorough surface shows only a slightly increased viability (Figure 10b).

#### 3.4.3. Comparison

The mNSCs are well adhered on all the untreated carbon surfaces, indicating good biocompatibility. We observed a clear difference on cell behavior on structured ta-C compared to structured PyC. On pyrolytic carbon, neither the cell number nor morphology seem to be affected by the different surface topographies, and cells distribution is rather homogeneous on all surfaces. On the contrary, the MTT reading for the cells grown on 2 μm ta-C pillars is higher compared to the planar reference surface. Moreover, on ta-C cell aggregation is evident and cell viability rate is increased on 2 μm pillars, especially on hexa-grid and random-grid topographies. It is unclear why specifically these two layouts provided clearly different results. Especially the difference between the two ordered structures, square-grid and hexa-grid, is particularly interesting because the pillar spacing is the same in both, but the angle to adjacent pillars changes from 90° to 60°. Also, in the hexa-grid the density of pillars is slightly higher compared to square-grid, which may support cellular movement and survival.

**Figure 11:**
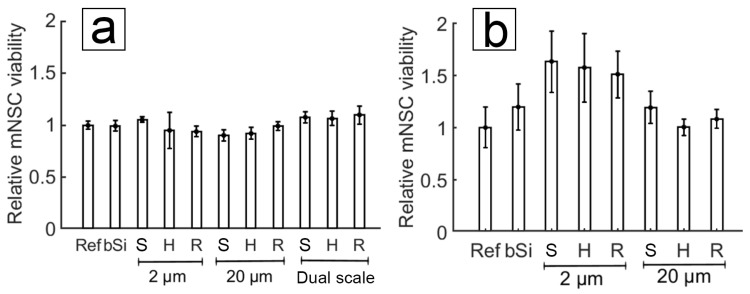
MTT assay readings for mNSC cultured on untreated carbon surfaces. Relative mNSC viability results on (**a**) PyC shows only minor differences between different structures, whereas (**b**) on ta-C the cell viability is increased with 2 μm pillars. The sample codes denote ref for reference, bSi for nanorough black silicon surface, S for square-grid, H for hexa-grid, and R for random-grid.

We have compared the viability of mNSCs on reference PyC and ta-C samples. The viability rate is higher on untreated ta-C compared to untreated PyC. The comparative study of relative mNSC viability is shown in Figure 11 for both materials. The graphs are not directly comparable in respect to cell numbers, as seeding of the cells was done in separate experiments.

We have also compared the viability of other cell types, neuroblastic PC-12 cells and C6 cells, on untreated reference PyC and ta-C samples. Also, with these the viability rate on ta-C is higher compared to PyC (data not shown). Even though PyC and ta-C are both carbon-based materials, they have several distinct characteristics that may result in different interphasal reactions and consequent cellular responses. Both thin films are extremely smooth: average roughness being 1.15 ± 0.14 nm for ta-C [11] and 0.9 ± 0.6 nm for PyC [19]. However, the surface chemical functionalities differ significantly: ta-C has significantly more oxygen functionalities (8.9%) than PyC (1.5%) [13]. The water contact angles were: (static) ta-C 67.4° ± 2.0° [57] and (dynamic) PyC advancing 86° ± 2° and receding 38° ± 3° [19].

The ability to guide cell clustering is significant for several cell processes. For example, control over cell aggregation is needed during embryonic body (EB) formation from embryonic stem cells and EB formation is a common method for producing different cell lineages for further applications. Further control of the cell clustering can also be exploited. For example, high density EB cultures lead to an increase of cardiomyocytes production without the addition of any cardiogenic growth factors [58]. In general, cell condensation is the pivotal stage in the development of skeletal and other mesenchymal tissues [59].

Although mNSCs generally require poly-l-lysine (PLL) coating for adhesion, we have previously observed that mNSC adhesion on ta-C is particularly good and MTT measured from mNSCs is similar on ta-C with and without PLL coating (not shown). On the other hand, on SU-8-based PyC, the number of cells (C6 glial cells, PC12-Adh neuroblastic cells and mNSCs) is slightly increased subsequent to surface oxidation and/or PLL coating [19]. All cell growth experiments in this study were done without PLL or any other coatings highlighting the good biocompatibility of both types of carbon.

Studies related to cell responses on micro/nanometric patterns have also been carried out by other research groups. Bugnicourt et al. [60] fabricated silicon pillar surface with submicrometer pillar diameter, and studied the effect on primary hippocampal neurite elongation and axonal differentiation. Their surface was coated with PLL, and they report that the nanometric scale influences many aspects of neuronal growth, but mostly accelerated neurite elongation. Britland et al. [61] studied the effect of two conflicting cues on cell fate. They had laminin as a chemical cue and grooves as topographical cue, both patterned on fused silica microscope slides. The cues were oriented orthogonally, and groove depth was changed in different experiments. The results showed that when the groove depth was 500 nm or smaller, cells (rat dorsal root nerve cells) reacted more to the chemical cues, but when the groove depth increased it started to dominate the orientation of cells. At 5 μm depth the topography oriented roughly 80% of the cell and laminin 7%. The interesting point with the study by Britland et al. is the fact that only by changing the depth (and not pitch or groove lateral size) they saw difference in cell morphology. Similar result is gained by Kaivosoja et al. [62] concluding that the height differences of the surface cause stretching of the cell cytoskeleton and regulate osteogenesis. With some cells, a chemical cue might be needed to activate the cell’s reaction to topographical cues [29,34]. All these results indicate the importance of surface topography and geometry to cell fate, but as demonstrated by our experiments, it is not the only factor that affects the cell behavior.

## 4. Conclusions

Three types of micro- and nanopillar surfaces with PyC or ta-C chemistry were fabricated in 20 μm, 2 μm and <1 μm length scales. Both materials provide comparable micro- and nanotopography for cell response. We showed that cells were able to adhere to the different structures on both carbon types. Interestingly, there was a clear difference on cell behavior on ta-C compared to PyC: viability of cells was unaffected by structures on PyC, whereas on ta-C surfaces the 2 μm features increased cell count and aggregation of cells. On the other hand, applying PLL coating on ta-C surface did not affect cell response whereas on PyC it slightly increased cell count, highlighting the different roles of surface geometry and chemistry.

In this work we have shown how to fabricate micro- and nanoscale rough, high surface area, structures from two carbon materials. Such surfaces are promising for applications that benefit from large active surface area to lower the impedance of electrodes, affect cell differentiation and viability and to increase sensitivity of electrochemical sensors.
